# Comparative Ability of Various Immunosuppressants as Adjuvants on the Activity of T1D Vaccine

**DOI:** 10.3390/vaccines12101117

**Published:** 2024-09-29

**Authors:** Xinyi Wang, Mengxin Xie, Tengjiao Li, Jiandong Shi, Meini Wu, Shihan Zhang, Jing Sun, Yunzhang Hu

**Affiliations:** 1 Institute of Medical Biology, Chinese Academy of Medical Sciences and Peking Union Medical College, Kunming 650118, China; wxyvicky@yeah.net (X.W.); oho_teemo@yeah.net (M.X.); litengjiao@stu.ynu.edu.cn (T.L.); shijiandong@imbcams.com.cn (J.S.); nini5440@163.com (M.W.); zsh15226927851@163.com (S.Z.); 2 School of Life Sciences, Yunnan University, Kunming 650091, China

**Keywords:** type 1 diabetes, adjuvants, GAD65 vaccine

## Abstract

**Background:** Type 1 diabetes (T1D) is an autoimmune disorder characterised by the destruction of insulin-producing beta cells in the pancreatic islets, resulting from a breakdown in immunological tolerance. Currently, T1D treatment primarily relies on insulin replacement or immunosuppressive therapies. However, these approaches often have significant drawbacks, including adverse effects, high costs, and limited long-term efficacy. Consequently, there is a pressing need for innovative immunotherapeutic strategies capable of inducing antigen-specific tolerance and protecting beta cells from autoimmune destruction. Among the various antigens, β-cell antigens like 65 kDa glutamic acid decarboxylase (GAD65) have been explored as vaccine candidates for T1D. Despite their potential, their effectiveness in humans remains modest, necessitating the use of appropriate adjuvants to enhance the vaccine’s protective effects. **Methods:** In this study, we evaluated the therapeutic potential of kynurenine (KYN), dexamethasone (DXMS), tacrolimus (FK506), and aluminium hydroxide (Alum) in combination with the GAD65 phage vaccine as adjuvants. **Results:** Our findings demonstrate that KYN, when used in conjunction with the GAD65 vaccine, significantly enhances the vaccine’s immunosuppressive effects. Compared to dexamethasone, FK506, and Alum adjuvants, KYN more effectively reduced the incidence and delayed the onset of T1D, preserved β-cell function, and promoted the induction of regulatory T cells and antigen-specific tolerance. These results suggest that KYN combined with vaccines could offer superior preventive and therapeutic benefits for T1D compared to existing treatments. Additionally, we investigated the dose-dependent effects of the GAD65 vaccine by including a low-dose group in our study. The results indicated that reducing the vaccine dose below 10^10^ plaque-forming units (pfu) did not confer any protective advantage or therapeutic benefit in combination with KYN. This finding underscores that 10^10^ pfu is the minimum effective dose for the GAD65 vaccine in achieving a protective response. In conclusion, KYN shows considerable promise as an adjuvant for the GAD65 vaccine in T1D therapy, potentially offering a more effective and durable treatment option than current immunosuppressive strategies.

## 1. Introduction

T1D is an autoimmune disorder marked by the destruction of insulin-producing β cells in the pancreatic islets, resulting from a loss of immunological tolerance. This process is driven by CD4^+^ and CD8^+^ effector T cells (Teff) that react against β-cell antigens [1]. As of 2021, approximately 8.4 million people worldwide are affected by T1D. According to the World Diabetes Federation (WDF), the prevalence is expected to surge to between 13.5 and 17.4 million by 2040, representing a 60–107% increase from 2021 [2,3]. The use of insulin replacement therapy has been fundamental in the management of T1D since the early 1920s. However, this approach does not address the underlying autoimmune response nor prevent long-term complications. Additionally, lifelong dependence on insulin imposes significant financial and psychological burdens on patients. Consequently, there is an urgent need for safe, long-lasting alternatives with minimal side effects. Immunotherapy is emerging as a promising strategy to prevent and potentially reverse the autoimmune assault on beta cells.

Recent advances in immunotherapy for T1D include the depletion of specific cell subsets using antibodies. For example, the anti-CD3 monoclonal antibody Teplizumab targets T cells, and the anti-CD20 monoclonal antibody Rituximab targets B cells. These treatments have shown promise in preserving C-peptide levels and delaying disease onset in the short term [4,5,6]. Notably, Teplizumab has received approval from the U.S. Food and Drug Administration (FDA) as a biologic therapy for T1D [7,8]. However, its high cost limits accessibility, and its effects may not be sustained in all patients when used alone.

An effective immunotherapeutic vaccine for autoimmune diseases like T1D should aim to suppress the inflammatory immune response driving the disease and redirect it towards promoting the proliferation and differentiation of antigen-specific regulatory T (Treg) cells. GAD65 is a significant autoantigen in T1D, and studies have shown that vaccines based on GAD65 protein or plasmid DNA can prevent and mitigate T1D in non-obese diabetic (NOD) mice [9,10]. While these vaccines have demonstrated efficacy in animal models, their success in humans has been limited. To enhance the effectiveness of these vaccines, adjuvants can be used to boost the immune response to the target antigen without eliciting a significant response to the adjuvant itself. Combining autoantigens with adjuvants can amplify the immune suppression mediated by the autoantigen, potentially overcoming the limited efficacy observed in human trials. One such vaccine, the GAD65-alum vaccine named Diamyd (developed by Diamyd Medical, Stockholm, Sweden), is currently in Phase III clinical development (NCT05018585, accessed on July 2021).

Immunosuppressive drugs are widely used in treating allergies, and autoimmune diseases, and preventing transplant rejection [11,12,13,14,15,16]. Despite their efficacy, these drugs typically provide only temporary suppression and do not induce lasting changes in immune responses against pathogenic recall antigens. Some immunosuppressive agents have been found to promote the transformation of natural T cells into regulatory T cells with suppressive functions in vitro. This suggests that using immunosuppressants as vaccine adjuvants could induce antigen-specific immune tolerance, offering a novel approach to vaccine design for autoimmune diseases.

Dexamethasone, a long-acting glucocorticoid, inhibits immune system overactivity and protects pancreatic β-cells from autoimmune destruction. FK506, also known as tacrolimus, is a potent immunosuppressant derived from Streptomyces species that primarily inhibits interleukin-2 (IL-2) release and T lymphocyte function. Studies have shown that combining FK506 and dexamethasone with insulin or other therapies can delay or reverse T1D progression, reduce insulin requirements, and improve glycaemic control [17,18,19,20]. Dexamethasone enhances T cell apoptosis, reduces T cell infiltration and activation, and promotes the differentiation and function of Tregs, thereby maintaining immune tolerance.

Kynurenine (KYN), a metabolite of tryptophan, is produced via the enzymatic action of tryptophan 2,3-dioxygenase (TDO) and indoleamine 2,3-dioxygenase (IDO). KYN binds to the aryl hydrocarbon receptor (AHR), modulating immune homeostasis [21,22]. AHR activation can directly suppress Teff function or enhance Treg induction by binding to the Foxp3 promoter [23,24]. Our previous studies [25] have indicated that KYN can serve as an effective immunosuppressive adjuvant for GAD65 T1D vaccines.

In this study, we compared the therapeutic efficacy of KYN in combination with the currently widely used immunosuppressants dexamethasone, FK506, and Alum, which have been used in combination with GAD65 in clinical trials, to evaluate the effect of these adjuvants on phage GAD65 vaccine. To explore the potential dose-sparing effects of KYN, we included a low-dose group of the phage GAD65 vaccine (L-GAD65) and a combination group (L-GAD65 + KYN). Our results indicate that KYN, as an immunosuppressant, was most effective and enhanced the immune responses to the T1D GAD65 phage vaccine by inducing regulatory T cells and antigen-specific tolerance. Compared to the commonly used adjuvants and immunosuppressants, KYN proved to be more effective in reducing the incidence and delaying the onset of T1D, as well as preserving β-cell function. Furthermore, when examining the low-dose group, we observed that a dose of 1 × 10^10^ pfu was required to achieve a protective effect in combination with KYN. The lower dose of 1 × 10^9^ pfu did not produce a sufficient therapeutic response, underscoring the importance of the vaccine dose in achieving optimal immunosuppressive efficacy. These findings highlight KYN’s potential as a novel adjuvant for the prevention and treatment of T1D, offering insights that could lead to more effective and durable therapeutic options.

## 2. Materials and Methods

### 2.1. Animal Immunisation and Assessment of Diabetes

The female NOD mice, aged 5 weeks, were obtained from GemPharmatech Co., Ltd. (Nanjing, China). All the animals were kept in a controlled environment with regulated light and temperature. The animal studies, including the euthanasia procedure, were carried out in accordance with the regulations and guidelines of the Institute of Medical Biology, Chinese Academy of Medical Sciences institutional animal care. They were also conducted following the AAALAC and IACUC guidelines. 

The mice were divided into nine groups in a random manner, each group consisting of 10 animals. They were subcutaneously immunised with the GAD65 phage vaccine (from our lab) alone or co-immunised with kynurenine (KYN) (Sigma, K8625, Saint Louis, MO, USA), dexamethasone (DXMS) (Sigma, D4902, USA), tacrolimus (FK506) (MCE, HY-13756A, Monmouth Junction, NJ, USA), or aluminium hydroxide (Alum) (from our lab) at weeks 6 and 8. The blank group was immunised subcutaneously with PBS, and the control group was immunised subcutaneously with an empty phage vaccine. The dose for the GAD65 group was 10^10^ pfu; the dose for the low-dose group (L-GAD65) was 10^9^ pfu. Table 1 shows the detailed groupings and doses.

**Figure 1:**
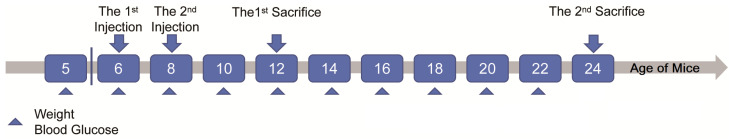
Immunisation procedures in mice and time points for body weight and blood glucose measurements. Two subcutaneous immunisations were performed at week 6 and week 8, and the animals were sacrificed at the 12th week and the 24th week to obtain eyeball blood, pancreas, and spleen.

The animal immunisation schedule and detection programme are shown in Figure 1. Blood was collected from the tail every two weeks to measure blood glucose levels using OneTouch Ultra Easy^®^ glucometer (Johnson & Johnson, New Brunswick, NJ, USA). Mice were diagnosed with diabetes if their blood glucose level was higher than 10.3 mmol/L and symptoms including polydipsia, polyphagia, polyuria, and weight loss occurred. Briefly, mice were sacrificed at weeks 12 and 24, and splenocytes and pancreases were harvested at different time points.

Relevant blood samples were obtained, and the sera were kept at a temperature of −20 °C until they were tested.

### 2.2. Antibodies and Synthetic Peptide

Fluorescently labelled monoclonal antibodies against mice, including anti-mouse CD4-PE-Cy5.5 (RM4-5), anti-mouse CD25-PE (PC61.5), anti-mouse FOXP3-APC (FJK-16s), anti-mouse CD80-APC (16-10A1), anti-mouse CD11c-PE (N418), and anti-mouse IL-10-FITC (JESS-16E3), were purchased from eBioscience. Fluorescently labelled anti-mouse monoclonal antibodies, including anti-mouse IL-10-PC7 (JES5-16E3) and anti-mouse TGF-β-FITC (TW7-16B4), were purchased from BioLegend. For immunofluorescence, the primary antibody INS Rabbit PolyAb and the CoraLite488-conjugated goat anti-rabbit secondary antibody were both purchased from Proteintech. The specific synthetic peptide acid sequences for GAD65 were TYEIAPVFVLLEYVT, EYVTLKKMREIIGWPGGSGD, KKGAAALGIGTDSVI, ALGIGTDSVILIKCDERGK, and TLEDNEERMSRLSK.

### 2.3. Histology and Immunofluorescence

Mouse pancreases were harvested at weeks 12 and 24 and immobilised in 4% paraformaldehyde. Tissue paraffin sections were prepared by Servicebio (Wuhan, Hubei, China) and stained with haematoxylin and eosin. The level of insulitis was assessed based on the following criteria: absence of cell infiltration (no insulitis), infiltration only at the periphery of the islets (peri-insulitis), less than 50% of the islet area infiltrated (mild insulitis), and 50% or more of the islet area infiltrated (severe insulitis) [26].

Insulin secreted by pancreatic was analysed by immunofluorescence. The paraffin sections were deparaffinized with ethanol and then closed with 1% BSA. Incubated with primary antibody INS Rabbit PolyAb (Proteintech, Rosemont, IL, USA) at 4 °C overnight, followed by washing in PBS, incubation with secondary antibodies and DAPI was then carried out for 10 min at RT avoiding the light. The images were captured using a digital scanner that has panoramic MIDI capabilities (3DHISTECH, Budapest, Hungary).

### 2.4. Flow Cytometry Analysis of Treg and DC Populations

Splenocytes were collected from mice at weeks 12 and 24. The spleen lymphocytes were rinsed with 1640 medium supplemented with 10% FBS and adjusted to a concentration of 1 × 10^6^/mL. The cells were incubated for 48 h at 37 °C with 5% CO_2_, with 100 μL of a GAD65 peptide pool containing 5 μg/mL of each individual peptide as previously described. For DCs, cells were stained with anti-CD11c-PE (N418) and anti-CD80-APC (16-10A1), followed by permeabilisation and staining with the intracellular anti-IL10-FITC (JESS-16E3) fluorescent-labelled monoclonal antibody. For Tregs, cells were stained with anti-CD4-PE-Cy5.5 (RM4-5) and anti-CD25-PE (PC61.5). After permeabilisation, samples were stained with intracellular anti-FOXP3-APC (FJK-16s), anti-IL-10-PC7 (JES5-16E3), and anti-TGF-β-FITC (TW7-16B4) fluorescent-labelled monoclonal antibodies. Following two washes, samples were resuspended in Permeabilisation Buffer and immediately analysed on a CytoFLEXS Flow Cytometer (BECKMAN COULTER, Brea, CA, USA). The flow cytometry data were analysed using CytExpert software 2.3.1.22. Flow staining kits were obtained from the Intracellular Fixation and Permeabilisation Buffer set and the FOXP3/Transcription Factor Staining Buffer Set (eBioscience, San Diego, CA, USA).

### 2.5. RNA Isolation and qPCRC

RNA was extracted using the Trizol method. qPCR samples were prepared with the GoTaq^®^ qPCR Master Mix kit (Promega, Madison, WI, USA) and detected using a real-time quantitative PCR instrument (Bio-Rad, Hercules, CA, USA). The primers used were as follows: IL-10: Forward: GCTCTTACTGACTGGCATGA; Reverse: CGCAGCTCTAGGAGCATGTG. TGF-β: Forward: CTAATGGTGGAAACCCACAACG; Reverse: TATCGCCAGGAATTGTTGCTG. GAPDH: Forward: TCAAGAAGGTGGTGAAGCAG; Reverse: AAGGTGGAAGAGTGGGAGTTG. The qPCR response procedures were as follows: 95 °C for 5 min; 38 cycles of (95 °C for 15 s; 55 °C for 15 s; 72 °C for 15 s). The melting curve was set at 65 °C to 95 °C, with an increment of 0.5 °C per cycle for 5 s.

### 2.6. Statistical Analyses

The data were reported as means ± standard deviations (SDs), and statistical analysis was conducted using the SPSS Graphpad Prism 8.0.2. A *p*-value less than 0.05 was deemed to be statistically significant.

## 3. Results

### 3.1. Effects of Different Vaccine Groups on NOD Mice

**Figure 2:**
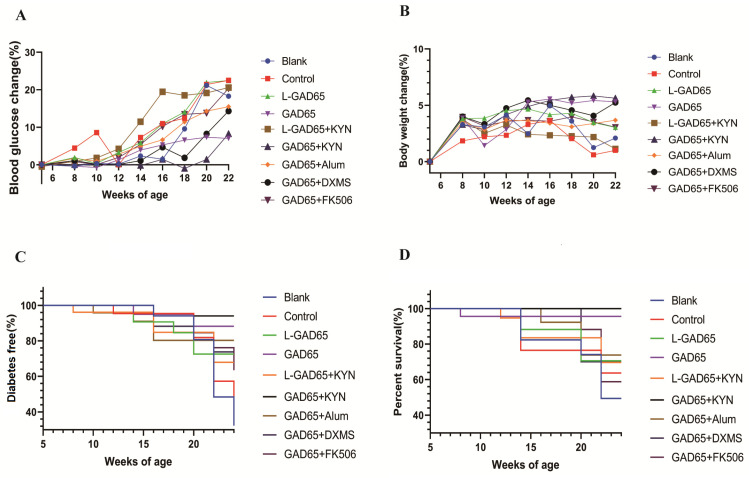
GAD65 phage vaccine + KYN inhibits hyperglycaemia and blocks the onset of autoimmune diabetes in NOD mice (n = 10). (**A**,**B**) NOD mice blood glucose and weight change curve. Body weight and blood glucose were measured at 5, 6, 8, 10, 12, 14, 16, 18, 20, and 22 weeks. (**C**) The rate of diabetes free. The GAD65 group and the GAD + KYN group preventing the development of hyperglycaemia in 88% and 94% for at least 6 weeks. (**D**) The survival curves. There is no NOD mouse died in the GAD65 group and the GAD + KYN group.

To compare the vaccine efficacy in each group of NOD mice, we administered GAD65 phage vaccine premixed with KYN, FK506, DXMS, and Alum via subcutaneous injection. The immunisation and sample collection schedule are shown in Figure 1. The blood glucose levels and body weights of the NOD mice were measured at 5, 6, 8, 10, 12, 14, 16, 18, 20, and 22 weeks of age. Fasting NOD mice were considered diabetic when their blood glucose exceeded 10.3 mmol/L, a threshold used as an early diagnostic criterion for diabetes in mice. Our findings (Figure 2A,C) revealed that disease progression in the blank and control groups was similar following the onset of diabetes, with onset occurring at week 16 in both groups and the incidence reaching up to 51.6% and 42.8%, respectively, by week 22. The addition of the adjuvants DXMS, FK506, and Alum did not enhance the vaccine effect but rather attenuated it compared to the GAD65 group. Additionally, there was a significantly reduced protective effect in the low-dose vaccine group, with a 22.2% decrease in L-GAD65 compared to GAD65 and a 38.6% decrease in L-GAD65 + KYN compared to GAD65 + KYN. However, both the GAD65 group and the GAD65 + KYN group exhibited substantial protection against hyperglycaemia onset in NOD mice, with 88% and 94% prevention rates, respectively, for at least 6 weeks.

Weight loss is an important symptom of T1D. Our results showed (Figure 2B) that the blank, control, GAD65 + FK506, L-GAD65, and L-GAD65 + KYN groups all exhibited signs of weight loss, while the GAD65 + Alum group showed less variability in weight change. Conversely, mice in the GAD65, GAD65 + KYN, and GAD65 + DXMS groups showed normal weight gain. Furthermore, survival data (Figure 2D) indicated that by week 24, the survival rates of mice were as follows: blank group 49.5%, control group 63.53%, and GAD65 + FK506 group 58.66%, and there were similar survival rates for the GAD65 + DXMS and GAD65 + Alum groups at approximately 74.34%. The survival rate of the GAD65 group was notably high at 95.24%, with no mortality observed in the GAD65 + KYN group, surpassing even that of the GAD65 group.

### 3.2. Preventive Effects of Various Vaccine Groups on Pancreatic Islet Inflammation in NOD Mice

Haematoxylin-eosin staining was performed on paraffin-embedded pancreatic sections to determine the level of inflammation in each group.

**Figure 3:**
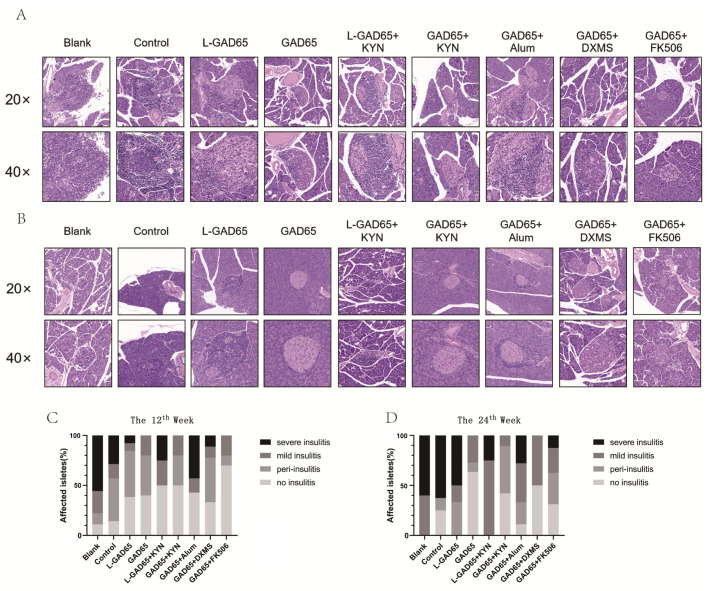
GAD65 phage vaccine + KYN suppresses islet inflammation. Mice pancreases were harvested at week 6 and 18, made into paraffin sections and stained with haematoxylin and eosin. (**A**) H&E staining of pancreatic tissue at the 12th week with a magnification of 20/40 (n = 5). (**B**) H&E staining of pancreatic tissue at the 24th week with a magnification of 20/40 (n = 5). (**C**) Pancreatic inflammation score at the 12th week. (**D**) Pancreatic inflammation score at the 24th week.

At the 12th week (Figure 3A,C), more than 55% of the blank group had developed severe insulitis, and over 30% of the control group exhibited severe insulitis. The L-GAD + KYN group was similar to the control group, and the L-GAD65 group showed a small amount of severe insulitis. The GAD65 group did not exhibit severe insulitis, whereas the GAD65 + Alum group had a high inflammation level, with over 45% of the mice displaying severe insulitis. The GAD65 + DXMS group had a small amount of severe insulitis, while the GAD65 + KYN and GAD65 + FK506 groups showed no severe insulitis. At the 24th week (Figure 3B,D), the blank and control groups exhibited more than half severe insulitis compared to the 12th week. The L-GAD65 and L-GAD65 + KYN groups showed a significant increase in the ratio of insulitis. The GAD65 + Alum and GAD65 + DXMS groups had a lower proportion of severe insulitis due to the mortality of severely affected mice, which were not included in the count. However, there was a slight increase in the proportion of insulitis. The GAD65 + FK506 group demonstrated a slight increase in mild and severe insulitis proportions, although these remained low. No cases of severe insulitis were observed in the GAD65 and GAD65 + KYN groups, with a higher proportion showing no signs of insulitis.

### 3.3. Protective Effects of Different Vaccine Groups on β-Cells and Insulin Secretion in NOD Mice

**Figure 4:**
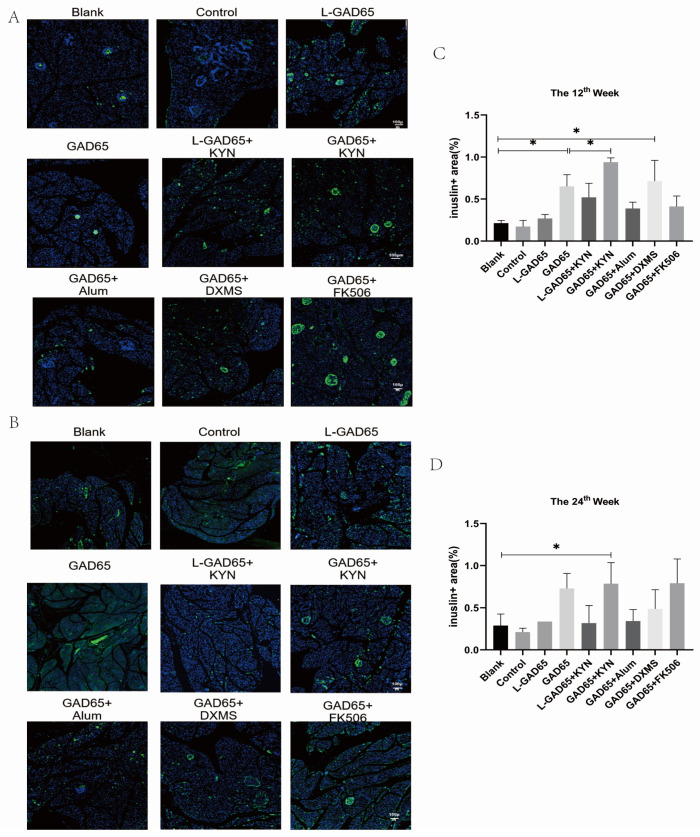
GAD65 phage vaccine + KYN inhibits islet β-cell apoptosis and protects insulin secretion. The pancreas of NOD mice was stained with insulin antibody (green), and all nuclei were labelled with DAPI (blue). (**A**) Immunofluorescence results at the 12th week. (**B**) Immunofluorescence results at the 24th week. (**C**) Relative area of insulin at the 12th week (n = 5, * *p* < 0.05). (**D**) Relative area of insulin at the 24th week (n = 5, * *p* < 0.05).

Immunofluorescence results at week 12 (Figure 4A,C) revealed that while pancreatic beta cells were predominantly preserved, there were notable differences in insulin secretion among the mice in different groups. Insulin secretion was notably low in the blank, control, and L-GAD65 groups. Mice in the L-GAD65 + KYN, GAD65 + Alum, and GAD65 + FK506 groups exhibited slightly higher levels of insulin secretion compared to the control group; however, these differences did not reach statistical significance. The insulin secretion levels in the GAD65, GAD65 + KYN, and GAD65 + DXMS groups were significantly higher than those in the blank group. Additionally, the insulin secretion level in the GAD65 + KYN group was significantly higher compared to the GAD65 group. By week 24 (Figure 4B,D), all groups exhibited varying degrees of reduction in islet β-cell area and a substantial decrease in insulin secretion. However, only the GAD65, GAD65 + KYN, and GAD65 + FK506 groups maintained relatively high levels of insulin secretion. Notably, only the insulin secretion level in the GAD65 + KYN group showed a significant difference from that in the blank group.

### 3.4. Differences in the Ability of Different Vaccine Groups to Inhibit DC Maturation

**Figure 5:**
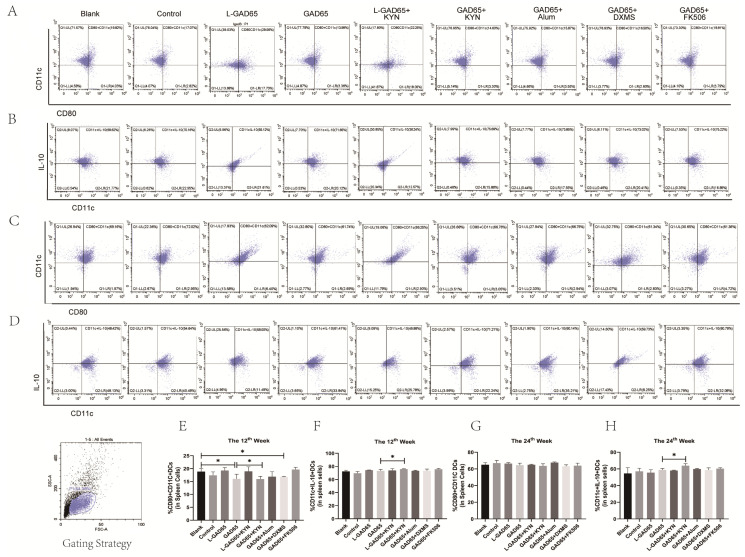
GAD65 phage vaccine + KYN can suppress dendritic cell maturation. Mice spleen lymphocytes were collected and the proportion of DC and IL-10 secreting DC was detected by flow cytometry. (**A**,**B**,**E**,**F**) The proportion of mature DC and IL-10 secreted by DC in spleen of NOD mice in each group at the 12th week (n = 5, * *p* < 0.05). (**C**,**D**,**G**,**H**) The proportion of mature DC and IL-10 secreted by DC in spleen of NOD mice in each group at the 24th week (n = 5, * *p* < 0.05).

Spleen lymphocytes were isolated at weeks 12 and 24 and subsequently stimulated with GAD65 peptide. Flow cytometry was used to detect the expression of CD80 and CD11c on dendritic cells (DCs), as well as IL-10 secretion by DCs. As shown in Figure 5A,E, at week 12, the percentage of mature DCs (CD80^+^CD11c^+^) in the L-GAD65 and L-GAD65 + KYN groups was higher compared to the blank group. The percentage of mature DCs (CD80^+^CD11c^+^) in the GAD65 + Alum group was lower than in the blank group but did not reach statistical significance. The GAD65 + FK506 group exhibited results similar to the blank group. Additionally, the percentage of mature DCs (CD80^+^CD11c^+^) in the GAD65, GAD65 + KYN, and GAD65 + DXMS groups was significantly lower than in the blank group, with the GAD65 + KYN group showing a notable decrease compared to the GAD65 group alone. However, these inhibitory effects did not persist until week 24, as shown in Figure 5C,G.

At week 12 (Figure 5B,F), no significant differences in IL-10 secretion by DCs were observed between most groups and the blank group, except for the GAD65 + KYN group, which showed significantly higher levels compared to both the blank group and the GAD65 group. Furthermore, the elevated IL-10 secretion in the GAD65 + KYN group was sustained until week 24 (Figure 5D,H).

### 3.5. Differences in the Ability of Various Vaccine Groups to Induce GAD65-Specific CD4^+^CD25^+^Foxp3^+^ Regulatory T Cells (Tregs)

Foxp3^+^ Tregs suppress the proliferation of effector T cells and promote immune tolerance both in vitro and in vivo. To evaluate the potential induction of Treg cells in NOD mice by different vaccine groups, spleen lymphocytes were isolated at weeks 12 and 24 and re-cultured with GAD65 peptide. The cells were then stained with anti-CD4-PE-Cy5.5, anti-CD25-PE, and anti-FOXP3-APC antibodies for flow cytometry analysis.

**Figure 6:**
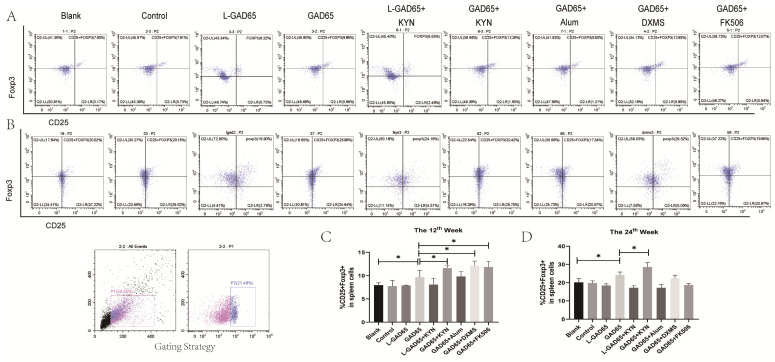
Significantly increased frequency of CD4^+^CD25^+^Foxp3^+^ Tregs in splenic lymph nodes of vaccine-immunised mice. Mice spleen lymphocytes were collected, and the proportion of CD4^+^CD25^+^Foxp3^+^ Tregs was detected by flow cytometry. (**A**,**C**) The proportion of CD4^+^CD25^+^Foxp3^+^ Tregs in spleen of NOD mice in each group at the 12th week (n = 5, * *p* < 0.05). (**B**,**D**) The proportion of CD4^+^CD25^+^Foxp3^+^ Tregs in spleen of NOD mice in each group at the 24th week (n = 5, * *p* < 0.05).

As shown in Figure 6A,C, at week 12, the proportion of CD4^+^CD25^+^Foxp3^+^ Tregs within the CD4^+^ T cell population in the control, L-GAD65, and L-GAD65 + KYN groups was similar to that in the blank group. Although the GAD65 + Alum group exhibited a higher proportion compared to the blank group, this difference was not statistically significant. The GAD65 group had a significantly higher proportion of Tregs than the blank group, and the proportions in the GAD65 + KYN, GAD65 + DXMS, and GAD65 + FK506 groups were all higher than in the GAD65 group. The elevated expression of CD4^+^CD25^+^Foxp3^+^ Tregs persisted until week 24 in both the GAD65 and GAD65 + KYN groups, with the GAD65 + KYN group showing a significantly higher proportion compared to the GAD65 group alone (Figure 6B,D). In contrast, no significant differences were observed in the other groups by week 24.

### 3.6. Differences in the Ability of Various Vaccine Groups to Induce Immune Tolerance in NOD Mice

**Figure 7:**
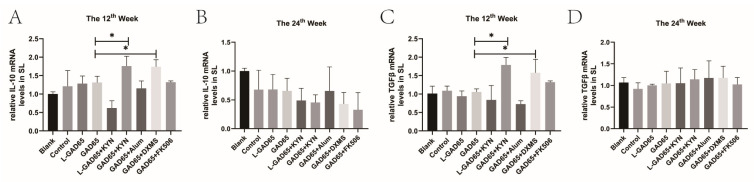
GAD65 phage vaccine + KYN and DXMS induced increased secretion of IL-10 and TGF-β in NOD mice. Mice spleen lymphocytes were collected, and the proportion of IL-10 and TGFβ secreted was detected by qPCR. (**A**,**C**) The proportion of IL-10 and TGF-β in spleen of NOD mice in each group at the 12th week (n = 5, * *p* < 0.05). (**B**,**D**) The proportion of IL-10 and TGF-β in spleen of NOD mice in each group at the 24th week (n = 5, * *p* < 0.05).

IL-10 and TGF-β play crucial roles in immune tolerance. Splenocytes were isolated at weeks 12 and 24, RNA was extracted, and real-time PCR was performed to assess the relative expression of IL-10 and TGF-β. At week 12, both the GAD65 + KYN and GAD65 + DXMS groups exhibited significantly higher IL-10 and TGF-β levels in the spleen compared to the GAD65 group (Figure 7A,C). However, this increased secretion was not sustained until week 24 (Figure 7B,D), with no significant differences observed between other groups and the blank group. 

To further investigate the secretion of IL-10 and TGF-β by regulatory T cells, splenic lymphocytes were isolated and cultured with GAD65 peptide at weeks 12 and 24. Subsequently, the cells were stained with anti-CD4-PE-Cy5.5, anti-CD25-PE, anti-IL-10-PC7, and anti-TGF-β-FITC antibodies and then analysed by flow cytometry.

**Figure 8:**
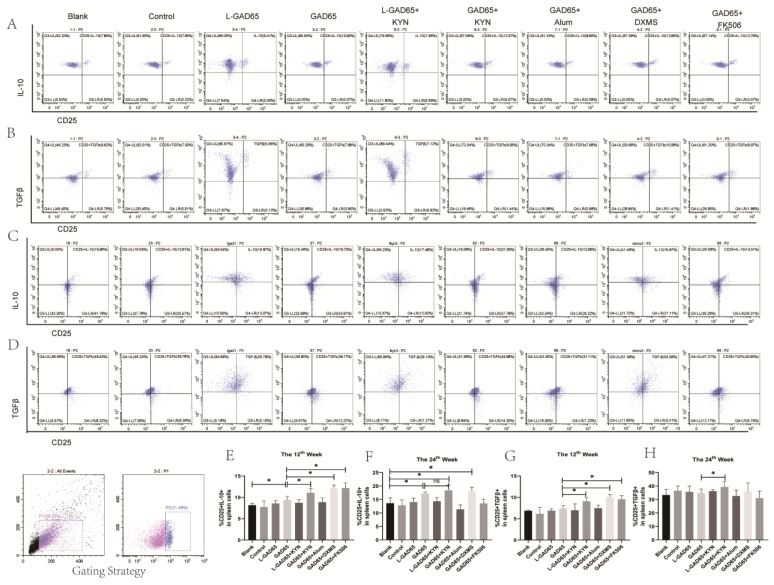
GAD65 phage vaccine + KYN induces immune tolerance in NOD mice. Mice spleen lymphocytes were collected, and the proportion of IL-10 and TGF-β secreted by Treg was detected by flow cytometry. (**A**,**B**,**E**,**G**) The proportion of IL-10 and TGFβ secreted by Treg in spleen of NOD mice in each group at the 12th week (n = 5, * *p* < 0.05). (**C**,**D**,**F**,**H**) The proportion of IL-10 and TGF-β secreted by Treg in spleen of NOD mice in each group at the 24th week (n = 5, * *p* < 0.05).

At week 12 (Figure 8A,B,E,G), the level of IL-10 and TGF-β produced by the CD4^+^CD25^+^ T cell population in the control group, the two low-dose groups, and the GAD65 + Alum group did not show significant differences compared to those in the blank group. Notably, GAD65 induced a higher proportion of IL-10 in the CD4^+^CD25^+^ T cell population than the blank group, and the proportion of IL-10 in the CD4^+^CD25^+^ T cell population of the GAD65 + KYN, GAD65 + DXMS, and GAD65 + FK506 groups (Figure 8A,E) was significantly higher than the GAD65 group. By week 24 (Figure 8C,F), only the proportion of IL-10 in the CD4^+^CD25^+^ T cell population of the GAD65, GAD65 + KYN, and GAD65 + DXMS groups was higher than the blank group, but there were no significant differences in the GAD65 + KYN and GAD65 + DXMS groups from the GAD65 group. Furthermore, at week 12 (Figure 8B,G), the GAD65 + KYN, GAD65 + DXMS, and GAD65 + FK506 groups produced a higher proportion of TGF-β in the CD4^+^CD25^+^ T cell population compared to the GAD65 group. However, there was no significant difference between the levels produced by the GAD65 group and those from the blank group. Additionally, only the induction effect of GAD65 + KYN persisted until 24 weeks (Figure 8D,H), with no significant difference observed between other groups and the blank group at this time point. 

## 4. Discussion

Our previous studies have shown that [25] therapeutic strategies for T1D have been significantly advanced through the development of antigen-specific immunity approaches. Among these, GAD65-based vaccines have emerged as particularly promising for T1D prevention due to their high specificity, excellent tolerability, safety, and ease of use. Preclinical studies have demonstrated their effectiveness in preventing T1D in NOD mice [10,27,28,29]. However, translating these results into successful human clinical applications has proven challenging, with outcomes often falling short of expectations. Key factors influencing the efficacy of GAD-based vaccines in humans include the number of induced Tregs, vaccine dosage, immunisation schedule, and regimen. Previous studies have shown that KYN can serve as an effective suppressive adjuvant for type 1 diabetes vaccines. Nevertheless, further screening and comparison of additional clinically used immunosuppressants are needed to optimise the efficacy of T1D vaccines.

Notably, blood glucose monitoring data revealed that the GAD65 + KYN group effectively prevented the development of hyperglycaemia in 94% of mice for at least 6 weeks, compared to 88% in the GAD65 group. By 22 weeks, the GAD65 + KYN combination prevented T1D in 45.38% of NOD mice, surpassing the 39.42% prevention rate observed with the GAD65 vaccine alone. These results highlight KYN’s ability to enhance the protective effects of the GAD65 vaccine. In contrast, groups supplemented with FK506, DXMS, or Alum did not show significant improvements in morbidity, mortality, or body weight compared to the GAD65 group. Remarkably, the GAD65 + KYN group maintained a stable body weight and exhibited no mortality, whereas the FK506 group experienced a high mortality rate of 41.2%. However, a significantly reduced protective effect was observed in the low-dose vaccine groups, L-GAD65 and L-GAD65 + KYN. These findings suggest that combining KYN with an appropriate dose of the GAD65 phage vaccine offers a more effective strategy for reversing T1D.

Pancreatic histological sections and immunofluorescence results provide a direct assessment of the differential protective effects of the GAD65 phage vaccine with various adjuvants on NOD mice. Four weeks after the final immunisation, the islets of mice in the GAD65, GAD65 + KYN, and GAD65 + FK506 groups showed minimal inflammatory cell infiltration and retained a substantial number of β-cells. In contrast, other groups exhibited varying degrees of insulitis and severe islet inflammation. Notably, the GAD65 + Alum group demonstrated heightened inflammation, with over 45% of islets displaying severe insulitis, indicating that Alum was less effective in inducing immunosuppression. The L-GAD65 and L-GAD65 + KYN groups showed a significant increase in insulitis ratio, with no protective effect observed. The increased proportion of intact islets and reduced inflammation in the GAD65, GAD65 + KYN, GAD65 + DXMS, and GAD65 + FK506 groups underscores the sustained protective effect conferred by administering a high dose of the GAD65 phage vaccine in combination with these adjuvants.

We also investigated the effects of the GAD65 phage vaccine on various dendritic cell (DC) subsets and their role in modulating immune responses. DCs are crucial in orchestrating immune responses, with distinct subsets such as cDC1, cDC2, and pDC activating different T cell subsets, including Th1, Th2, and Treg, respectively [30]. Mature DCs generally activate T cell effector functions, whereas immature DCs (iDCs) promote T cell tolerance [31]. The immunosuppressive cytokines IL-10 and TGF-β, secreted by iDCs, play essential roles in maintaining immune tolerance and homeostasis [32]. Our findings demonstrated that the GAD65 phage vaccine, whether administered alone or in combination with KYN or DXMS, significantly suppressed the maturation of DCs. Notably, the GAD65 + KYN group exhibited a substantial reduction in mature DCs compared to the GAD65 group, highlighting KYN’s significant enhancement of the vaccine’s ability to inhibit DC maturation. However, this inhibition did not persist beyond 24 weeks.

The impact of KYN also extended to the modulation of cytokine secretion from DCs. The combination of the GAD65 phage vaccine and KYN significantly enhanced the secretion of IL-10 from DCs, with this effect lasting up to 24 weeks, indicating a prolonged immunosuppressive role of KYN. In contrast, although DXMS also stimulated IL-10 secretion, its effects were not sustained as long. These results suggest that KYN not only enhances but also prolongs the vaccine’s ability to promote a regulatory DC profile, which is crucial for maintaining immune tolerance in T1D.

The efficacy of the T1D vaccine relies on a mechanism that activates specific regulatory immune responses against disease-triggering antigens, thereby suppressing harmful effector T cell responses [33,34]. Regulatory Foxp3^+^ T cells can achieve an immunosuppressive effect through the production of TGF-β and IL-10, leading to the elimination of effector T cells via cytolysis [35]. Despite efforts to enhance the immunosuppressive effect of GAD65 vaccines, such as the advanced GAD65-alum vaccine Diamyd, which incorporates an aluminium hydroxide adjuvant, early-stage clinical trials yielded unsatisfactory results despite the induction of regulatory Foxp3+ T cells.

Four weeks after the final immunisation, both the GAD65 vaccine alone and its combinations with KYN, FK506, or DXMS induced GAD65-specific Foxp3^+^ Tregs. The KYN group maintained high levels of these Tregs and TGF-β up to 24 weeks, indicating a unique, durable, and protective effect associated with KYN. Additionally, while KYN, FK506, and DXMS all enhanced IL-10 secretion from Tregs, only KYN sustained this effect over the long term. These findings underscore KYN’s superior ability to induce and maintain a prolonged regulatory immune response in NOD mice. KYN activates the KYN-AhR signalling pathway, which primarily maintains immune tolerance by inducing the generation and function of Tregs [36], inhibiting the proliferation and differentiation of effector T cells to reduce the intensity of the immune response [37], upregulating the expression of the immune checkpoint molecule PD-1 to inhibit T cell activity upon binding to its ligands PD-L1 or PD-L2 [38], and promoting macrophage polarisation to the M2 type to suppress immune reactions [39].

IL-10 and TGF-β are pivotal immunoregulatory factors that play crucial roles in immune tolerance from multiple perspectives. IL-10 is a pleiotropic cytokine with the ability to exert both immunosuppressive and immunostimulatory effects on various cell types. It primarily inhibits the antigen presentation capacity of monocytes, macrophages, and dendritic cells, thereby reducing their activation of T cells and suppressing cell-mediated immune responses. Additionally, IL-10 suppresses the synthesis and release of inflammatory factors, downregulates MHC class II antigen expression, inhibits NK cell and CTL functions, and promotes the development of regulatory T cells [40,41]. TGF-β contributes to maintaining peripheral tolerance by promoting the differentiation of regulatory T cells and regulating mucosal immunity and autoimmunity through the differentiation of Th17 cells and the modulation of the Th17/Treg balance [42]. Our results demonstrated that the GAD65 phage vaccine, when supplemented with KYN or DXMS, could transiently stimulate the secretion of IL-10 and TGF-β. However, only KYN sustained this stimulation over the long term, particularly in promoting TGF-β secretion from GAD65-specific Tregs. The prolonged release of IL-10 and TGF-β suggests that the combination of the GAD65 phage vaccine and KYN effectively modulates the immune balance in T1D, providing enduring protection against the disease. In addition, this combination of vaccine and KYN for subcutaneous immunisation capitalises on the slower absorption kinetics associated with subcutaneous injection, potentially creating a sustained immunosuppressive microenvironment provided by KYN that is conducive to enhancing antigen-induced specific immune tolerance.

Despite these promising findings, our experiments also indicated that a low-dose GAD65 phage vaccine, with or without KYN, did not effectively prevent disease in NOD mice or induce antigen-specific immune tolerance. While low antigen doses can theoretically induce immune tolerance, excessively low concentrations may fail to achieve effective induction. Adjuvants play a critical role in enhancing vaccine efficacy, but they cannot compensate for an insufficiently potent vaccine. Therefore, a minimum dose of 1×10^10^ pfu is necessary to achieve the desired protective effect, underscoring the importance of vaccine dosage in conjunction with adjuvant selection.

Taken together, our study demonstrated that the combination of 1 × 10^10^ pfu GAD65 phage vaccine with KYN provided a long-lasting and superior protective effect compared to Alum, FK506, and DXMS.

In addition, efficacy and safety are the fundamental requirements and greatest challenges for any vaccine. A T1D vaccine must effectively protect pancreatic β cells, delay or reverse disease progression, lower blood glucose levels, and reduce complications, all while avoiding serious side effects. Our study has demonstrated promising results in an animal model, highlighting the potential of KYN as a key component in T1D vaccination strategies. Moving forward, further evaluation of kynurenine’s safety will be conducted to ensure its suitability as an adjuvant for vaccines.

## 5. Conclusions

Currently, clinical trials for T1D vaccines are incomplete, and more data and evidence are needed to establish their efficacy and safety. Key areas for future innovation include identifying new adjuvants that enhance immune tolerance while minimising side effects. Looking ahead, the development of vaccines for T1D not only holds promise for managing this specific autoimmune condition but also opens avenues for treating other autoimmune diseases. The principles learned from T1D vaccine research could be applied to create immunotherapies for a wide range of autoimmune disorders.

## Figures and Tables

**Table 1:** Grouping of animals, dose, and adjuvant.

Group	Note	Number	Dose	Adjuvant	Injection Times
Blank		10	PBS		2
Control	Empty phage	10	100 μL (1 × 10^11^ pfu/mL)		2
L-GAD65		10	100 μL (1 × 10^10^ pfu/mL)		2
GAD65		10	100 μL (1 × 10^11^ pfu/mL)		2
L-GAD65 + KYN		10	100 μL (1 × 10^10^ pfu/mL)	KYN (200 μg)	2
GAD65 + KYN		10	100 μL (1 × 10^11^ pfu/mL)	KYN (200 μg)	2
GAD65 + Alum		10	100 μL (1 × 10^11^ pfu/mL)	Al(OH)_3_ (300 μg)	2
GAD65 + DXMS		10	100 μL (1 × 10^11^ pfu/mL)	DXMS (10 μg)	2
GAD65 + FK506		10	100 μL (1 × 10^11^ pfu/mL)	FK506 (100 μg)	2

## Data Availability

The data presented in this study are available on request from the corresponding author.
